# Direct disk testing versus isolation and antimicrobial susceptibility testing of urine from urinary tract infection

**Published:** 2018-02

**Authors:** Raz Nawzad Mohammad, Sherko Ali Omer

**Affiliations:** 1 Department of Clinical Biochemistry, College of Pharmacy, University of Sulaimani, Sulaymaniyah, Iraq; 2 Department of Microbiology, College of Medicine, University of Sulaimani, Sulaymaniyah, Iraq

**Keywords:** Urinary tract infection, Antimicrobial, Direct sensitivity testing

## Abstract

**Background and Objectives:**

Urinary tract infections are common infections that can be caused by many bacterial pathogens. The susceptibility of such pathogens to antimicrobial agents is identified by different methods including disk diffusion test, direct sensitivity testing and determination minimum inhibitory concentration. The present study was conducted to isolate and identify bacteria cultured from urine samples and compare the results of direct sensitivity test (DST) against Kirby-Bauer’s disk diffusion antimicrobial sensitivity (AST) with respect to reliability, time and cost.

**Materials and Methods:**

Midstream urine samples were inoculated on blood and MacConkey agar plates; growth was evaluated after colony counting. We identified isolates based on their cultural and biochemical properties, and Vitek^®^ 2 system. Both DST and AST were performed on Mueller-Hinton agar using 10 antimicrobial agents. Error rate was calculated between the DST and AST as the proportion of comparisons between DST and AST test results. The comparisons represented as “very major error”, “major error”, or “minor error” and “agreement” (i.e, no error).

**Results:**

We tested 373 urine samples, of them 257 (68.9%) were from females and 116 (31.1%) from males. Primary cultivation detected growth (>10^5^ cfu/mL) from 206 (55.23%) samples; Gram-negative isolates were the most common isolates; these included *Escherichia coli* (111, 51.87%), and *Klebsiella pneumoniae* (19, 8.88%), while *Staphylococcus aureus* (14, 6.54%) was the main Gram-positive isolate. From the 1940 individual comparisons of DST and AST of single (pure) bacterial isolates, 12 comparisons (0.6%) represented very major errors, 9 (0.5%) major errors, 36 (1.8%) minor errors, and 1883 comparisons (97.1%) were in agreement.

**Conclusion:**

*E. coli* was the most common isolate. Cefixime and cefpodoxime were found to be the most ineffective antimicrobial agents, while meropenem and nitrofurantoin were the most effective agents against all isolated urinary pathogens. DST and AST almost give the same results in pure cultures, and direct antimicrobial susceptibility for urine specimens can safely replace standard antimicrobial susceptibility in urinary tract infection.

## INTRODUCTION

Urinary tract infections (UTIs) are infections involving in any part of urinary system and are among the most common infections affecting 150 million people worldwide annually (1). Females have a higher risk for UTIs than most males due to their urinary tract anatomy, and it is estimated that 40% to 50% of women experience at least one UTI in their life (2). Other risk factors for UTIs include conditions that may impede urine flow, such as enlarged prostate, congenital anomalies, urinary stones and strictures, and presence of urinary catheters (3).

The main causes of UTIs are bacteria, but fungi and parasites may also cause UTIs. Gram-negative *E. coli* bacterium is the most common uropathogenic bacterium causing UTIs (4). Others include *K. pneumoniae*, *P. aeruginosa* and *Proteus* spp., while Gram-positive bacteria such as *Enterococcus faecalis*, *S. aureus* and *Staphylococcus saprophyticus* have also been reported to cause UTIs (4).

Clinical manifestation of UTIs ranges from asymptomatic bacterial colonization of the bladder to irritative symptoms, such as frequency and urgency, which are associated with lower urinary tract infection, while upper urinary tract infection is associated with fever, chills, and flank pain (5). Microscopy of urine test can be used to determine pyuria and bacteriuria. Pyuria can be determined using centrifuged urine specimen (> 8 WBC/mL) (6). Bacteriuria is detected using Gram stain of uncentrifuged urine (7); however, its sensitivity in detecting UTIs with bacteria less than 10^5^ cfu/mL is low and may not detect infections with bacteria of 10^2^–10^3^ cfu/mL.

Urine culture is necessary to identify the infecting microorganisms. Cultures are necessary in nosocomial UTIs, recurrent UTIs, treatment failures, complicated, and serious UTIs (8). Significant bacteriuria means the presence of 10^5^ or more cfu/mL of urine. However, in catheterized patients and many patients with lower UTIs, colony counts lower than 10^5^ cfu/ mL are significant if the specimens are obtained by suprapubic aspirate or catheterization. Accordingly, the most appropriate diagnostic criterion for urine culture specimens obtained through suprapubic aspirate or catheterization is a bacterial concentration of equal to and greater than 10^2^ cfu/mL (8, 9). The VITEK^®^ 2 bacterial identification system can be used to identify different bacteria, but it needs initial bacterial cultures and tests before selecting the test cards.

Identifying urinary pathogens and their antimicrobial response provides information for an accurate management of UTI. Antimicrobial susceptibility test (AST) of isolated bacteria is usually tested by the Kirby-Bauer disk diffusion method (10). However, the results are only available after 48 to 72 hours after sampling, as bacteria need to be cultured first before AST can be performed.

Direct sensitivity testing (DST) of urine specimens offers a rapid and accurate method to determine antimicrobial susceptibility for acute UTI, particularly when the urine bacterial concentration is >10^5^ cfu/mL (11). While DST is performed directly on the urine sample by disk diffusion, it is potentially useful in the management of critically ill patients, as the time to achieve the result is shortened by approximately 24 hours (12). Direct sensitivity testing of urine is reliable in monobacterial Gram-negative infections; moreover, with an increase in antimicrobial resistance, DST can aid UTI management and reduce the use of broad-spectrum antimicrobials (13). Nevertheless, the American Society for Microbiology (ASM), the British Society for Antimicrobial Chemotherapy (BSAC), and the European Committee on Antimicrobial Susceptibility Testing (EUCAST) seriously criticize DST, as the inoculum is not standardized, and as it is less sensible to detect all bacteria present in the same sample. On the other hand, DST provides clinicians with an early microbiological information and permits tailored antibiotic use and a decrease in antimicrobial-related adverse events (12).

We conducted this study to isolate and identify the bacteria cultured from urine samples and to compare the results of direct sensitivity test against Kirby-Bauer disk diffusion antimicrobial sensitivity test with regards to reliability, time, and cost.

## MATERIALS AND METHODS

Midstream urine samples were collected from specimens submitted for urine culture to the public health laboratory in city of Sulaimani from December 2015 to June 2016. Urine samples were cultured within 2 hours after collection, otherwise the samples were stored at 4–6°C up to 4 hours.

Blood agar base (HIMEDIA^®^ laboratories, India), MacConkey agar and Muller Hinton agar (Liofilchem^®^, Italy) were prepared according to manufacturer’s recommendation. We inoculated 10 μL of urine sample onto each of blood and MacConkey agar plates. Agar plates were incubated at 35–37°C for 18 to 24 hours; if growth was detected, then, the colonies were counted visually. However, if growth was not detected, the culture plates were reincubated for additional 24 hours before the culture was considered as negative or no growth. A urine sample was considered as UTI if bacteria were isolated at a concentration of ≥10^5^ cfu/mL (11). We identified bacterial isolates based on their cultural and biochemical properties. Vitek^®^ 2 system (bioMérieux, France) was used with the ID-GNB card to identify Gram-negative bacilli from pure cultures.

We performed direct susceptibility test (DST) on all urine samples. A sterile cotton swab was dipped into well-mixed, unadjusted urine specimen and streaked onto a Mueller-Hinton agar plate in 3 directions. The agar plate was allowed to dry for 2 to 5 minutes, and we placed antimicrobial disks on the media and pressed them firmly onto the agar surface with sterile forceps. We read the results after incubation for 16 to 18 hours at 37^o^C. We also performed standard antimicrobial susceptibility (AST) using Kirby-Bauer disk diffusion method and by 0.5 Mc-Farland saline suspension of the isolate with the same antimicrobial disks used in DST (10, 11). We used the following antimicrobial disks (Bioanalyse^®^, Turkey): meropenem (MEM 10 μg/disk), nitrofurantoin (F 300 μg/disk), gentamicin (CN 10 μg/disk), amoxicillin-clavulanic acid (AMC 20/10 μg/disk), trimethoprime-sulphamethoxazole (SXT 25/ μg/disk), cefixime (CFM 5 μg/disk), cefuroxime (CXM 30 μg/ disk), cefpodoxime (CPD 10 μg/disk), ciprofloxacin (CIP 5 μg/disk), and levofloxacin (LEV 5 μg/disk). We followed the criteria provided by performance standards for antimicrobial susceptibility testing to determine isolates susceptibility and to evaluate the susceptibility results (14).

We calculated error rate between the DST and AST results as the proportion of comparisons between direct and standard test results. The comparisons represented the followings (15):

- Very major error (VMJ): A susceptible result by the direct method and a resistant result by the standard method
- Major error (MJ): A resistant result by the direct method and a susceptible result by the standard method
- Minor error (MN): Any discrepancy involving an intermediate susceptibility by one method and susceptibility or resistance by the other
- Agreement (A): Agreement or “no error” when both methods’ results agree using the respective criteria

We performed statistics using Stata Software, Version 13.

## RESULTS

From 373 urine samples, 257 (68.9%) were from females and 116 (31.1%) were from males (ratio 2.2:1). The females’ age ranged from 1 to 83 years (mean 33 years ± 17.3 SD), while males’ age ranged from 1 to 83 years (mean 38.5 years ± 21.2 SD). Most samples were from 51 to 60-year-old group (20.69%) males, and this age group had the most positive cultures (23%). The females’ urine samples were predominately from 31 to 40-year-old group (26.46%), and the urine from this age group had the most positive cultures (24%).

Primary cultivation of urine samples detected growth (>104 cfu/mL) in 206 (55.23%) samples, 52 (44.83%) of the male’s samples and 154 (59.92%) of the female’s samples, with a statistically significant relationship (*p*< 0.05).

From 206 positive cultures, 196 (95.145%) yielded a single isolate, while 10 samples (4.85%) showed mixed isolates. A total of 214 bacterial species were isolated, 170 (79.4%) Gram-negative bacteria and 44 (20.6%) Gram-positive bacteria. The main Gram-negative isolates were *E. coli* (111, 51.87%), *K. pneumoniae* (19, 8.88%), and *Proteus mirabilis* (14, 6.54%). The main Gram-positive isolates included 14 (6.54%) *S. aureus*, 10 (4.67%) *Staphylococcus saprophyticus*, 6 (2.8%) *Staphylococcus epidermidis*, and 9 (4.2%) *Enterococcus faecalis* (Table 1). Two fungal isolates were isolated and identified as *Candida albicans.*

**Table 1.** The isolated bacterial species from urine with UTI

Bacterial species*	**Number**	**Percentage**
*Escherichia coli*	111	51.87
*Klebsiella pneumoniae*	19	8.88
*Proteus mirabilis*	14	6.54
***Staphylococcus aureus***	14	6.54
***Staphylococcus saprophyticus***	10	4.67
***Enterococcus faecalis***	9	4.21
*Pseudomonas aeruginosa*	6	2.80
***Staphylococcus epidermidis***	6	2.80
*Enterobacter cloacae*	5	2.34
*Morganella morganii*	4	1.87
***Streptococcus pneumoniae***	3	1.40
*Enterobacter aerogenes*	2	0.93
*Serratia marcescens*	2	0.93
***Streptococcus pyogenes***	2	0.93
*Pseudomonas fluorescence*	1	0.47
*Salmonella enterica*	1	0.47
*Vibrio alginolyticus*	1	0.47
*Yersinia aldovae*	1	0.47
*Shigella sonnie*	1	0.47
*Sphingomonas sediminicola*	1	0.47
*Aeromonas salmonicida*	1	0.47
Total	214	100

* Gram-positive bacteria are bold type-faced.

Antimicrobial sensitivity (AST) of isolates showed that *E. coli* was resistant against cefixime (72.55%), followed by amoxicillin-clavulanic acid (69.6%), cefpodoxime (68.63%), trimethoprim-sulfamethoxazole (67.65%), and cefuroxime (59.8%). Meropenem was the most effective drug against *E. coli* isolates (98.04%), followed by nitrofurantoin (78.43%). The susceptibility pattern of *K. pneumoniae* showed resistance to trimethoprim-sulfamethoxazole (77.87%), followed by amoxicillin-clavulanic acid and cefpodoxime (66.67%), while they were sensitive to meropenem (88.9%), levofloxacin (72.22%), and gentamicin (55.5%). *S. aureus* isolates showed resistance to cefpodoxime and cefixime (63.64%), followed by trimethoprim-sulfamethoxazole (54.55%), while *S. aureus* was sensitive to meropenem (91%), nitrofurantoin (72.73%), and amoxicillin-clavulanic acid and gentamicin (63.64%) (supplementary data).

Regardless of the isolated bacterial species, we found that all the urinary isolates showed resistance to cefixime (74.23%), cefpodoxime (69.59%), trimethoprim-sulfamethoxazole (65.45%), cefuroxime (55.7%), ciprofloxacin (49.5%), and levofloxacin (48.5%), while sensitivity to meropenem was 91.2%, to nitrofurantoin was 63.9%, and to gentamicin was 52.1% (Table 2).

**Table 2.** Comparison of the bacterial response to 10 antimicrobial agents using direct antimicrobial sensitivity testing (DST) versus standard antimicrobial sensitivity testing (AST)

**Antimicrobial agents**	**Direct antimicrobial sensitivity**			**Standard antimicrobial sensitivity**			***p* value**

	**S (No.) %**	**I (No.) %**	**R (No.) %**	**S (No.) %**	**I (No.) %**	**R (No.) %**	
Meropenem	177 (91.2)	10 (5.2)	7 (3.6)	177 (91.2)	9 (4.6)	8 (4.1)	0.942
Amoxicillin-clavulanic acid	64 (33)	17 (8.76)	113 (58.24)	57 (29.38)	18 (9.28)	119 (61.34)	0.745
Trimethoprim-sulfamethoxazole	58 (29.9)	7 (3.6)	129 (66.5)	58 (29.9)	9 (4.6)	127 (65.45)	0.875
Gentamicin	102 (52.6)	7 (3.6)	85 (43.8)	101 (52.1)	10 (5.1)	83 (42.8)	0.756
Nitrofurantoin	129 (66.5)	9 (4.6)	56 (28.9)	124 (63.9)	14 (7.2)	56 (28.9)	0.552
Cefixime	46 (23.71)	5 (2.58)	143 (73.71)	46 (23.71)	4 (2.06)	144 (74.23)	0.944
Cefuroxime	54 (27.8)	32 (16.5)	108 (55.7)	53 (27.3)	33 (17)	108 (55.7)	0.987
Cefpodoxime	47 (24.23)	12 (6.18)	135 (69.59)	47 (24.23)	12 (6.18)	135 (69.59)	1.0
Ciprofloxacin	82 (42.3)	16 (8.2)	96 (49.5)	83 (42.8)	15 (7.7)	96 (49.5)	0.981
Levofloxacin	88 (45.4)	10 (5.1)	96 (49.5)	91 (46.9)	9 (4.6)	94 (48.5)	0.939

Response to antimicrobial agent; S: susceptible; I: intermediate response; R: resistance

Direct antimicrobial susceptibility testing of urine form UTI samples (>10^5^ cfu/mL) showed that the most effective agents were meropenem (91.2%), nitrofurantoin (66.5%), and gentamicin (52.6%), but resistance was observed against cefixime (73.71%), cefpodoxime (69.59%), and trimethoprim-sulfamethoxazole (66.5 %) (Table 2).

From the 1940 individual comparisons of DST and AST of single isolates, 12 (0.6%) represented VMJ errors, 9 (0.5%) MJ errors, and 36 (1.8%) MN errors, but 1883 (97.1%) were in agreement. Satisfaction according to antimicrobial agent showed marked differences in error rates between the antimicrobial agents, with error rates being the lowest for carbapenems, cephalosporins, fluoroquinolones, and nitrofurantoin. For *E. coli* (n=102), VMJ errors were 0.7% for all agents, MJ errors were 0.4%, and MN minor errors were found to be 1.3%. Most MJ errors were detected in interpretation of amoxicillin-clavulanic acid and maximum MN errors were found in trimethoprim-sulfamethoxazole (Table 3).

**Table 3.** Discrepancies in bacterial response to 10 antimicrobial agents using direct antimicrobial sensitivity testing (DST) versus standard antimicrobial sensitivity testing (AST)

**Antimicrobial agent**	**All organisms *n*=194**			***E. coli n*=102**			**Other Gram- negatives *n* =52**			**Gram-positive *n*=40**		

	**VMJ**	**MJ**	**MN**	**VMJ**	**MJ**	**MN**	**VMJ**	**MJ**	**MN**	**VMJ**	**MJ**	**MN**
Meropenem	0	0	3	0	0	1	0	0	2	0	0	0
Amoxicillin-clavulanic acid	5	0	9	4	0	3	0	0	4	1	0	2
Trimethoprim-sulfamethoxazole	1	1	8	1	1	5	0	0	1	0	0	2
Gentamicin	2	3	3	1	0	0	1	1	1	0	2	2
Nitrofurantoin	2	1	5	0	1	2	1	0	2	1	0	0
Cefixime	0	0	1	0	0	1	0	0	0	0	0	1
Cefuroxime	0	0	3	0	0	1	0	0	1	0	0	1
Cefpodoxime	1	1	2	1	1	0	0	0	1	0	0	1
Ciprofloxacin	1	1	1	0	0	0	0	0	1	1	1	0
Levofloxacin	0	2	1	0	1	0	0	0	1	0	1	0
Total (%)	12 (0.6)	9 (0.5)	36 (1.8)	7 (0.7)	4 (0.4)	13 (1.3)	2 (0.4)	1 (0.2)	14 (2.7)	3 (0.75)	4 (1)	9 (2.25)

* Discrepancies: VMJ: very major error; MJ: major error; MN: minor error

** Agreement for all organisms was 1883 (97.1%) out of 1940 comparisons.

## DISCUSSION

In this study, females were refereed for urine culture more than males (2.2:1 ratio), indicating that UTIfeatures are more common among females. Positive urine culture was also found more among females than in males urine samples, indicating that UTIs are more common in females. Different figures of UTIs were reported, but in all UTI was found to be more common in females, as females have higher risks for UTIs due to short, straight urethra (16).

We detect UTI in in 206 (55.23%) samples. Our results were higher than those reported from Northern and Southern India (35.1%) (17), Nigeria (39.69%) (18), and Italy (22.6%) (19). This variation may be due to differences in the environment, social habits, and the standard of personal hygiene.

In our study, the Gram-negative bacilli isolates were more than Gram-positive bacteria isolates. Gram-negative bacilli are major causative agents of UTI due to their predominance in gut flora, their pili and other structures (20). In our results, *E. coli* was the predominate isolate, as *E. coli is* the main urinary pathogen with different isolation rate, ranging from 48.6 to 67.6% (19, 21). Other studies reported higher figures of *E. coli* (80.5%) (22), while lower figures than our results have also been reported (23). Because we did not obtain clinical history, this difference was expected, as it was related to the difference in presentation or clinical UTI types.

In our study, Gram-positive isolates from urine were mostly staphylococci including 14 *S. aureus* isolates. Staphylococci were the main Gram-positive isolates from UTIs although others found that coagulase-negative staphylococci were more common in UTIs. *S. pneumoniae* is not commonly considered an agent of UTI. We reported 3 isolates out of all 206 positive urine cultures.

We have tested *in vitro* susceptibility of different isolates using both DST and AST methods. Management of UTI is complicated by increased prevalence of antibiotic resistance. Most of our isolates showed resistance to many antimicrobials including third generation cephalosporin, trimethoprim-sulfamethoxazole, and amoxicillin-clavulanic acid. These antimicrobials are used frequently for different infections in our community. This was also observed from different studies (24, 25), so emerging resistance is related to the high rate of antimicrobial usage.

The increasing frequency of the trimethoprim-sulfamethoxazole resistance is worrying, as this agent is being frequently prescribed for uncomplicated UTIs and for other infections in many developed and developing countries (23, 26). In this study, 65.45% resistance rate to trimethoprim-sulfamethoxazole was observed.

Among the quinolones we tested, resistance to ciprofloxacin was 49.5% and to levofloxacin was 48.5% by AST. Comparable findings have been reported for ciprofloxacin (26). A fluoroquinolone cross-resistance phenomenon may explain this finding since the both drugs also share an enzyme target (27).

Resistance in Gram-negative bacteria has been increasing, particularly in the past few years. This is mainly due to the spread of strains producing extended-spectrum β-lactamases (ESBLs), such as CTX-M enzymes or AmpC β-lactamases. Many of the isolates producing these enzymes are also resistant to trimethoprim-sulfamethoxazole, quinolones, and aminoglycosides, often due to plasmid co-expression of other resistance mechanisms (24).

The spectrum of UTI agents and resistance rates of uncomplicated community-acquired UTI, complicated UTI, and nosocomial-acquired UTI can differ substantially from region to region and over time. Resistance to antimicrobial agents can be readily transferred among bacteria by transmissible elements. There are many reasons for this alarming situation including rigorous marketing of antibiotics, inappropriate prescription of antibiotics without appropriate sensitivity testing, easy availability in the pharmacy, and poor infection control strategies (28).

In our study, DST showed accuracy of 97.1% when compared to AST in 194 pure cultures, and the results of DST were similar to those of AST and were obtained earlier than 24 hours. Numerous studies showed overall agreement between AST and DST on 90% of urine samples, so our study agrees with other studies (12, 13, 15, 29, 30). Test errors were more common in association with older antimicrobial agents, agents with a high prevalence of antimicrobial resistance, non-*E. coli* strains, low urine bacterial concentrations, mixed growth in the direct test, and the presence of multiple significant organisms in urine (15).

Of the 100 comparisons in mixed growth between DST and AST (supplementary data), 2% were VMJ errors, 2% MJ errors, and 18% were MN errors, while 78% were in agreement, so mixed growth in DST was associated with relatively increased error as reported previously (15).

For all bacteria isolates, errors were significantly more common and agreement was significantly less common for non-*E. coli* strains rather than for *E. coli* strains (15). In our study, the agreements were 97.6%, 96.7%, and 96% for *E. coli*, non-*E. coli*, and Gram-positive strains, respectively. We also documented antimicrobial agent-specific differences in error rates in DST, as have also been reported by other studies (12, 15). Traditional agents were often highly error prone, and newer agents were almost error free. It was evident that the aggregate performance of DST could be artificially raised or lowered by the choice of antimicrobial agents included in the study (15). In our study, the highest percentage of discordant results occurred in the β-lactam antibiotics amoxicillin-clavulanic acid (14 or 0.72%), and similar results have also been reported (12), this was followed by trimethoprim- sulfamethoxazole, gentamicin and nitrofurantoin in our study. There were few differences for cephalosporins, fluroquinolones, and meropenem, which were comparable with other studies (15, 30).

The main advantage of DST is that it offers the possibility of obtaining results 24 hours earlier than AST and will be more efficient if the organism is also identified with the same time frame. Early initiation of antibiotic treatment is essential for effective management of UTI to reduce morbidity, mortality, cost of treatment, and to prevent antibiotic resistance. On the other hand, DST has some potential drawbacks including the likelihood of a non-interpretable DST with low bacterial concentrations and ambiguities in mixed cultures that lead to higher error rates. Some isolates with similar properties can be easily considered as a single isolate, especially when present in small numbers. Another recognized problem with DST for urine specimens is the high proportion of negative tests if DST is done on all urine specimens, most of which are culture-negative or contain fewer than 10^5^ cfu/mL. DST of these types of specimens are both waste of time and of antimicrobial drug disks, so that the cost for this method is higher than AST after primary cultivation (12, 15).

We concluded that *E. coli* was the most common isolate from urine of UTI. Cefixime and cefpodoxime were found to be the most ineffective drugs against all our isolates, while meropenem and nitrofuran were the most effective. DST and AST tests in pure cultures almost give the same results, so DST in UTI can safely replace AST in UTI. Moreover, DST of urine is potentially useful in managing critically-ill patients, as the time required for sensitivity tests is shortened, and thus the use of empirical broad-spectrum antimicrobials agents is reduced. However, the overall cost may be higher if all submitted urine samples are tested rather than just performing primary isolation and AST on definite UTI samples only.

## References

[1] Stamm WE Norrby SR Urinary tract infections: disease panorama and challenges. J Infect Dis 2001;183 Suppl 1:S1–4. 11171002 10.1086/318850

[2] Foxman B Urinary tract infection syndromes: occurrence, recurrence, bacteriology, risk factors, and disease burden. Infect Dis Clin North Am 2014;28:1–13. 24484571 10.1016/j.idc.2013.09.003

[3] Hooton TM Bradley SF Cardenas DD Colgan R Geerlings SE Rice JC Diagnosis, prevention, and treatment of catheter-associated urinary tract infection in adults: 2009 international clinical practice guidelines from the infectious diseases society of America. Clin Infect Dis 2010;50:625–663. 20175247 10.1086/650482

[4] Flores-Mireles AL Walker JN Caparon M Hultgren SJ Urinary tract infections: epidemiology, mechanisms of infection and treatment options. Nat Rev Microbiol 2015;13:269–284. 25853778 10.1038/nrmicro3432 PMC4457377

[5] Pietrucha-Dilanchian P Hooton TM Diagnosis, treatment, and prevention of urinary tract infection. Microbiol Spectr 2016;4(6). doi: 10.1128/microbiolspec.UTI-0021-2015. 28087935

[6] Hiraoka M Hida Y Hori C Tsuchida S Kuroda M Sudo M Urine microscopy on a counting chamber for diagnosis of urinary infection. Acta Paediatr Jpn 1995;37:27–30. 7754761 10.1111/j.1442-200x.1995.tb03680.x

[7] Carroll KC Hale DC Von Boerum DH Reich GC Hamilton LT Matsen JM Laboratory evaluation of urinary tract infections in an ambulatory clinic. Am J Clin Pathol 1994;101:100–103. 7506476 10.1093/ajcp/101.1.100

[8] Wilson ML Gaido L Laboratory diagnosis of uri-nary tract infections in adult patients. Clin Infect Dis 2004;38:1150–1158. 15095222 10.1086/383029

[9] Stark RP Maki DG Bacteriuria in the catheterized patient. What quantitative level of bacteriuria is relevant? N Engl J Med 1984;311:560–564. 6749229 10.1056/NEJM198408303110903

[10] Bauer A Kirby W Sherris J Turck M Antibiotic susceptibility testing by a standardized single disk method. Am J Clin Pathol 1966;45:493–496. 5325707

[11] Collee JG Miles RS Watt B Tests for the identiication of bacteria. In: Collee JG Fraser AG Marmion BP Simmons A, editors. In Mackie and Mc Artney Practical Medical Microbiology London, Churchill-Livingstone, UK: 1996 p. 433.

[12] Coorevits L Boelens J Claeys G Direct susceptibility testing by disk diffusion on clinical samples: a rapid and accurate tool for antibiotic stewardship. Eur J Clin Microbiol Infect Dis 2015;34:1207–1212. 25698312 10.1007/s10096-015-2349-2 PMC4426127

[13] Breteler KB Rentenaar RJ Verkaart G Sturm PD Performance and clinical significance of direct antimicrobial susceptibility testing on urine from hospitalized patients. Scand J Infect Dis 2011;43:771–776. 21696255 10.3109/00365548.2011.588609

[14] CLSI Performance standards for antimicrobial disk susceptibility tests: twenty third informational supplement. Wayne, PA: Clinical and laboratory standards institute 2013.

[15] Johnson JR Tiu FS Stamm WE Direct antimicrobial susceptibility testing for acute urinary tract infections in women. J Clin Microbiol 1995;33:2316–2323. 7494020 10.1128/jcm.33.9.2316-2323.1995 PMC228402

[16] Abejew AA Denboba AA Mekonnen AG Prevalence and antibiotic resistance pattern of urinary tract bacterial infections in Dessie area, North-East Ethiopia. BMC Res Notes 2014;7:687. 25280498 10.1186/1756-0500-7-687 PMC4195856

[17] Gupta S Kapur S Padmavathi DV Comparative prevalence of antimicrobial resistance in community-acquired Urinary Tract Infection Cases from Representative states of northern and southern India. J Clin Diagn Res 2014 9;8(9):DC09–12. 10.7860/JCDR/2014/9349.4889 PMC4225884 25386432

[18] Oladeinde BH Omoregie R Olley M Anunibe JA Urinary tract infection in a rural community of Nigeria. N Am J Med Sci 2011;3:75–77. 22540069 10.4297/najms.2011.375 PMC3336890

[19] Magliano E Grazioli V Deflorio L Leuci AI Mattina R Romano P Gender and age-dependent etiology of community-acquired urinary tract infections. Scientific World Journal 2012;2012:349597. 22629135 10.1100/2012/349597 PMC3351074

[20] Nash Anthony A. Dalziel Robert G. Fitzgerald J. Ross MIMS’ pathogenesis of infectious diseases 6 ed Great Britain: Elsevier; 2015 356 p.

[21] Dimitrov TS Udo EE Emara M Awni F Passadilla R Etiology and antibiotic susceptibility patterns of community-acquired urinary tract infections in a Kuwait hospital. Med Princ Pract 2004;13:334–339. 15467308 10.1159/000080470

[22] Raka L Mulliqi-Osmani G Berisha L Begolli L Omeragiq S Parsons L Etiology and susceptibility of urinary tract isolates in Kosova. Int J Antimicrob Agents 2004;23 Suppl 1:S2–5. 15037322 10.1016/j.ijantimicag.2003.09.009

[23] Hamawandi AM Omer SA Tayib TH Mustafa MK Antibacterial susceptibility in urinary tract infection among children in Sulaimani. JSMC 2015;5:1–6.

[24] Pallett A Hand K Complicated urinary tract infections: practical solutions for the treatment of multiresistant gram-negative bacteria. J Antimicrob Chemother 2010;65 Suppl 3:iii25–33. 20876625 10.1093/jac/dkq298

[25] Dibua UME Onyemerela IS Nweze EI Frequency, urinalysis and susceptibility profile of pathogens causing urinary tract infections in Enugu State, southeast Nigeria. Rev Inst Med Trop Sao Paulo 2014;56:55–59. 24553609 10.1590/S0036-46652014000100008 PMC4085832

[26] Shariff V A AR Shenoy MS Yadav T M R The antibiotic susceptibility patterns of uropathogenic *Escherichia coli*, with special reference to the fluoroquinolones. J Clin Diagn Res 2013;7:1027–1030. 23905095 10.7860/JCDR/2013/4917.3038 PMC3708190

[27] Odongo CO Anywar DA Luryamamoi K Odongo P Antibiograms from community-acquired uropathogens in Gulu, northern Uganda--a cross-sectional study. BMC Infect Dis 2013;13:193. 23627344 10.1186/1471-2334-13-193 PMC3643887

[28] Nandihal Namratha W Profile of urinary tract infection and quinolone resistance among *Escherichia coli* and *Klebsiella species* isolates. IJCMAS 2015;4: 749–756.

[29] Kallenius G Dornbusch K Hallander HO Jakobsson K Comparison of direct and standardized antibiotic susceptibility testing in bacteriuria. Chemotherapy 1981;27:99–105. 7471907 10.1159/000237963

[30] Oakes AR Badger R Grove DI Comparison of direct and standardized testing of infected urine for antimicrobial susceptibilities by disk diffusion. J Clin Microbiol 1994;32:40–45. 8126202 10.1128/jcm.32.1.40-45.1994 PMC262966

